# FadA promotes DNA damage and progression of *Fusobacterium nucleatum*-induced colorectal cancer through up-regulation of chk2

**DOI:** 10.1186/s13046-020-01677-w

**Published:** 2020-09-29

**Authors:** Pin Guo, Zibin Tian, Xinjuan Kong, Lin Yang, Xinzhi Shan, Bingzi Dong, Xueli Ding, Xue Jing, Chen Jiang, Na Jiang, Yanan Yu

**Affiliations:** 1 Department of Neurosurgery, The Affiliated Hospital of Qingdao University, Qingdao, 266003 People’s Republic of China; 2 Department of Gastroenterology, The Affiliated Hospital of Qingdao University, No. 16, Jiangsu Road, Qingdao, 266003 Shandong Province People’s Republic of China; 3 Department of Endocrinology and Metabolism, The Affiliated Hospital of Qingdao University, Qingdao, 266003 People’s Republic of China

**Keywords:** Colorectal cancer, *Fusobacterium nucleatum*, FadA, chk2, DNA damage, E-cadherin/β-catenin pathway

## Abstract

**Background:**

Globally, colorectal cancer (CRC) affects more than 1 million people each year. In addition to non-modifiable and other environmental risk factors, *Fusobacterium nucleatum* infection has been linked to CRC recently. In this study, we explored mechanisms underlying the role of *Fusobacterium nucleatum* infection in the progression of CRC in a mouse model.

**Methods:**

C57BL/6 J-Adenomatous polyposis coli (APC) Min/J mice [APC (Min/+)] were treated with *Fusobacterium nucleatum* (10^9^ cfu/mL, 0.2 mL/time/day, i.g., 12 weeks), saline, or FadA knockout (FadA−/−) *Fusobacterium nucleatum*. The number, size, and weight of CRC tumors were determined in isolated tumor masses. The human CRC cell lines HCT29 and HT116 were treated with lentiviral vectors overexpressing chk2 or silencing β-catenin. DNA damage was determined by Comet assay and γH2AX immunofluorescence assay and flow cytometry. The mRNA expression of chk2 was determined by RT-qPCR. Protein expression of FadA, E-cadherin, β-catenin, and chk2 were determined by Western blot analysis.

**Results:**

*Fusobacterium nucleatum* treatment promoted DNA damage in CRC in APC (Min/+) mice. *Fusobacterium nucleatum* also increased the number of CRC cells that were in the S phase of the cell cycle. FadA−/− reduced tumor number, size, and burden in vivo. FadA−/− also reduced DNA damage, cell proliferation, expression of E-cadherin and chk2, and cells in the S phase. Chk2 overexpression elevated DNA damage and tumor growth in APC (Min/+) mice.

**Conclusions:**

In conclusion, this study provided evidence that *Fusobacterium nucleatum* induced DNA damage and cell growth in CRC through FadA-dependent activation of the E-cadherin/β-catenin pathway, leading to up-regulation of chk2.

## Background

Colorectal cancer (CRC) is associated with somatic mutational and epigenetic events affecting tumor development and the host immune system [1]. As a disease affecting the digestive tract from the colon to the rectum, CRC typically starts with polyps in the digestive tract which gradually enlarge, attract blood vessels, and become metastatic to spread to other tissues [2]. As a major global health challenge [3], CRC is ranks 3rd for incidence but 2nd in terms of mortality on a global scale [4]. The overall cure rate of CRC has not been improved significantly in Asia in the last decade, the five-year survival rate remains at around 60%, and although the survival time has risen in recent years, the mortality rate remains high [5]. In addition to non-modifiable risk factors such as age [6, 7], personal history of inflammatory bowel disease or adenomatous polyps [8], family history of CRC [9], ingestion of food that contains carcinogenic compounds [10], lack of physical activity [11], cigarette smoking, and heavy alcohol consumption [12, 13] have been demonstrated to contribute to CRC development. These risk factors suggest that both prevention and treatment of CRC are equally important.

*Fusobacterium nucleatum* may be a newly discovered environmental risk factor for CRC [14]. *Fusobacterium nucleatum* was first reported in CRC tissue in 2011, linking this oral bacterium to this disease [15]. Later, *Fusobacterium nucleatum* was reported to be associated with CRC in Chinese patients [16]. *Fusobacterium nucleatum* has been also linked to other human diseases including periodontal diseases, pregnancy disorders, appendicitis, cardiovascular disease, rheumatoid arthritis, and respiratory tract infections [17]. Although the etiology of *Fusobacterium nucleatum*-induced CRC is not completely understood, many studies showed that microbial imbalance and infection are believed to be the main factors [18, 19]. Nevertheless, the molecular mechanisms that are involved in *Fusobacterium nucleatum*-induced CRC remain to be fully elucidated.

The E-cadherin/β-catenin complex is important to the integrity of epithelial cells [20]. This complex has been mechanistically linked to the progress of various cancers, including gastric cancer [21], glioblastoma [22], and *Fusobacterium nucleatum*-induced CRC [23]. FadA, a novel adhesin of the periodontal pathogen *Fusobacterium nucleatum*, consists of two forms, pre-FadA and mature FadA (mFadA), constituting a functional FadA complex (FadAc) [24]. Studies have suggested that *Fusobacterium nucleatum* may cause CRC by inducing inflammation and suppressing host immunity [25], possibly through modulating the E-cadherin/β-catenin pathway via FadA adhesion in *Fusobacterium nucleatum* [26–28]. Checkpoint kinase 2 (Chk2) is a multifunctional enzyme that has been shown to be central to cell cycle arrest and apoptosis by DNA damage [29]. Based on these previous findings, we further investigated the involvement of the E-cadherin/β-catenin pathway and FadA adhesion in *Fusobacterium nucleatum*-induced CRC that involved DNA damage induced by a common mediator chk2 in a mouse model.

## Materials and methods

### Ethics statement

All animal experiment protocols were approved by the Institutional Animal Ethics Committee of the Affiliated Hospital of Qingdao University. Great efforts were made to minimize the numbers, suffering and pain of the included animals.

### APC ^min/+^ mouse model

C57-APC (Min/+) knockout mice were established from C57BL/6 J-Adenomatous polyposis coli (APC) Min/J mice and propagated to a total of 40 mice. Mice were kept in a specific pathogen-free (SPF) facility Animal Research Center. Three days before intragastric administration of *Fusobacterium nucleatum*, streptomycin (2 mg/mL) was added to drinking water to ensure consistent microflora and promote colonization of *Fusobacterium nucleatum*. APC (Min/+) mice (*n* = 20) were treated with wild-type (WT) *Fusobacterium nucleatum* [10^9^ cfu/mL, 0.2 mL/time/day in sterile phosphate buffered saline (PBS), i.g., 12 weeks]. Another group of APC (Min/+) mice (*n* = 10) were treated with FadA-knockout *Fusobacterium nucleatum* US1 (FadA−/− *Fusobacterium nucleatum*) (10^9^ cfu/mL, 0.2 mL/time/day in sterile PBS, i.g., 12 weeks). Mice in the complete negative control (NC) group (*n* = 10) received intragastric administration of sterile PBS (0.2 mL/time/day, 12 weeks). The body weight and growth of mice were observed weekly. After treatment, the mice were euthanized under anesthesia with pentobarbital sodium at 40 mg/kg, followed by recording of tumor measurements and histopathological analysis. The tumor tissues were cut longitudinally and measured. The number of tumors was calculated and the size (diameter) of the tumors was quantified as < 1 mm, 1–2 mm, 2–3 mm, or greater than 3 mm. The algorithm used for tumor burden was the sum of each tumor diameter.

### Bacterial strains

*Fusobacterium nucleatum* was purchased from American Type Culture Collection (ATCC, Manassas, VA, USA; #25586). WT *Fusobacterium nucleatum* and FadA−/− *Fusobacterium nucleatum* were cultured in Columbia blood agar with 5 μg/mL heme, 5% desalted sheep blood, and 1 μg/mL vitamin K1 (Sigma-Aldrich, St. Louis, MO, USA) in a 37 °C anaerobic glove box containing 85% N_2_, 10% H_2_ and 5% CO_2_ [30]. *Escherichia coli* (MG1655, ATCC, Manassas, VA, USA) was propagated in Luria Bertani medium (BD Biosciences, Franklin Lakes, NJ, USA) at 37 °C in an aerobic incubator.

### Cell culture and infection

HT29 and HCT116 cells (ATCC, Manassas, VA, USA) were cultured in McCoy’s 5A media (#16600082, Thermo Fisher scientific, Waltham, MA, USA) containing 10% fetal bovine serum (FBS). Lentiviral vector pLVX-EFGL overexpressing chk2 (RuiChuBio, Shanghai, China), and lentiviral vectors (pLKO.1-puro) encoding short hairpin RNA (sh)-β-catenin and sh-negative control (NC) (Sigma-Aldrich, Darmstadt, Germany) were packaged by GenePharma (Shanghai, China). Upon achieving 80% confluence, cells were added with 5 μL lentivirus (10^8^ TU) for infection.

### Cell proliferation assay

HT29 and HCT116 cells were seeded in a 24-well plate at 1 × 10^4^ cells/well and added with 2 mL complete medium. The cells were treated with WT *Fusobacterium nucleatum*, FadA−/− *Fusobacterium nucleatum* (multiplicity of infection [MOI] = 100 or 1000), or *Escherichia coli* for 2 h and also treated with Protein tyrosine kinase (PTK) inhibitor genistein (50 mM, S1628, Beyotime Biotechnology, Shanghai, China) for 1 h. Cells treated with sterile PBS were used as complete NC. Cell counts were performed at 6h, 24h and 48 h using a hemocytometer. Each experiment was repeated 3 times.

### Tumor xenograft experiment

HT29 or HCT116 cells were co-cultured with WT or FadA−/− *Fusobacterium nucleatum*, *Escherichia coli* MG1655 (MOI: 1000:1) or PBS for 24 h. Then, the cells were washed three times with PBS and collected after trypsin treatment. The cell suspension was then mixed with WT or FadA−/−*Fusobacterium nucleatum*, *Escherichia coli* or PBS at a MOI of 20:1 and injected into the right flank (100 μL/mice, s.c.) of 6-week-old male nude mice (BALB/c, *n* = 5/group, Shanghai Academy of Sciences, Shanghai, China). After 3 h of subcutaneous injection, the mice were injected with piperacillin (150 mg/kg, i.p.) to kill the bacteria. Nude mice were raised under SPF conditions and provided with food and water normally. The tumor size was measured every 5 days, and tumor volume (Vol) was calculated as follows: Vol = 1/2 (length × width^2^). Nude mice were euthanized 35 days later, with tumors excised and weighed. The tissues were rapidly frozen in liquid nitrogen and stored at − 80 °C.

### Immunohistochemical and immunofluorescence staining

Sections of xenograft tumor tissues or CRC tissues were dewaxed, rehydrated, and boiled in citrate buffer for antigen extraction and blocking. Tissues were then incubated with primary rabbit antibodies to Ki-67 (1:500, ab15580, Abcam, Cambridge, UK) and primary mouse antibodies to proliferating cell nuclear antigen (PCNA, 1:300, m0879, Dako, Carpinteria, CA, USA), β-catenin (1:2000, ab6302, Abcam, Cambridge, UK), and chk2 (1:200, ab47433, Abcam, Cambridge, UK). The sections were observed under a fluorescence microscope (Zeiss, Thornwood, NY, USA).

Tissue sections prepared on glass glides were washed three times with PBS (3 min/time) in the plate. Sections were fixed with 4% paraformaldehyde for 15 min and washed three times with PBS for 3 min each. Tissues were blocked by 1 × PBS containing 3 mg/mL bovine serum albumin (BSA), 100 mM glycine, and 0.25% Triton X-100 for 30 min. The tissues were then probed with primary rabbit antibodies (Abcam, Cambridge, UK) against β-catenin (1:1000, ab22656) and chk2 (1:200, ab47433) at 4 °C. Thereafter, the sections were washed with PBS (three times, 5 min each) and incubated with fluorophore-bound Alexa Fluor® 594 secondary antibody (1:1000, ab150120, Abcam, Cambridge, UK) or Alexa Fluor® 488 (1:1000, AB150077, Abcam, Cambridge, UK) at room temperature for 1 h. Nuclei were stained with 4′,6-diamidino-2-phenylindole (DAPI). The sections were then washed with PBS (three times, 5 min each), soaked in distilled water, and air-dried. These sections were then observed under a FV-1000 confocal microscope.

### Comet assay (single cell gel electrophoresis)

Comet assay was performed using a Trevigen Comet Assay™ kit (Trevigen, Gaithersburg, MD, USA), according to the manufacturer’s instructions. In brief, HCT116 and HT29 cells were seeded at 1 × 10^5^ cells/well in tissue culture plates, and serum starved with 2% FBS-reduced medium overnight (16–18 h). Cells were co-cultured with WT or FadA−/− *Fusobacterium nucleatum*, *Escherichia coli* (MOI: 1000:1 or 100:1), or PBS for 24 h. The cells were washed three times with PBS and collected after trypsin treatment. Cell concentration was adjusted to 1 × 10^5^ cells/mL, mixed with 1% L-mannose (low melting agarose, Trevigen, Gaithersburg, MD, USA) at 37 °C, and loaded to 20-well slide provided from the Comet assay. Slides were placed in a pre-cooled lysis solution at 4 °C for 60 min and then treated with an alkaline electrophoresis solution (300 mM NaOH, 1 mM ethylene diamine tetraacetic acid [EDTA], pH > 13) at room temperature for 20 min in the dark. The slides were then transferred to pre-cooled fresh alkaline electrophoresis solution and electrophoresed at 21 V using Comet Analytical Electrophoresis System II (Trevigen, Gaithersburg, MD, USA) for 30 min and washed twice in dH_2_O for 5 min each and with 70% ethanol for 5 min. The slides were stained with 50 μL SYBR™ Gold nucleic acid gel (1: 10,000 in Tris-EDTA solution, S-11494, Thermo Fisher scientific, Waltham, MA, USA) for 30 min in the dark and observed under a Leica DM6000B upright microscope.

### γH2AX formation determined by immunofluorescence assay

HCT116 and HT29 cells were seeded in an 8-well slide system at 5 × 10^4^ cells/well and serum starved in 2% FBS-reduced medium overnight. The cells were co-cultured with WT or FadA−/−*Fusobacterium nucleatum*, *Escherichia coli* (MOI: 1000:1 or 100:1), or PBS for 24 h, washed with cold PBS and fixed in 3.7% aldehyde-free methanol (Thermo Fisher scientific, Waltham, MA, USA) for 30 min on ice. The cells were permeabilized with ice-cold methanol for 10 min, washed with PBS to remove methanol, and blocked with PBS containing 1% BSA and 5% goat serum for 1 h on ice. They were next incubated with phosphorylated H2AX histone antibodies (1:400, ab2893, Abcam, Cambridge, UK) overnight at 4 °C, washed with PBS, and incubated with Alexa Fluor 647-labeled goat anti-rabbit Immunoglobulin G (IgG) (H + L) antibody (Life Technologies, Carlsbad, CA, USA) for 45 min at room temperature. Then, the cells were washed with PBS, mounted with Vectashield mounting medium with DAPI (VectorLabs, Burlingame, CA, USA), and observed under a Leica DM6000B upright microscope.

### Flow cytometry

HCT116 and HT29 cells were co-cultured with WT or FadA−/− *Fusobacterium nucleatum*, *Escherichia coli* (MOI: 1000:1 or 100:1), or PBS for 24 h (flow cytometry) or 48 h (cell cycle assay). After co-culture, the cells were collected in PBS, fixed in 1% methanol-free cold formaldehyde solution (Thermo Fisher scientific, Waltham, MA, USA) for 15 min, washed in PBS, and incubated overnight at − 20 °C in 70% ethanol. They were next washed with PBS containing 1% BSA and 0.2% Triton X-100 (BSA-T-PBS), and incubated with anti-H2AX-phosphorylated (Ser139) antibody (1:200 diluted in TPBS, BioLegend, San Diego, CA, USA) labeled with Alexa Fluor 647 at 4 °C overnight. The cells were then washed with BSA-T-PBS and incubated with propidium iodide (Life Technologies, Carlsbad, CA, USA) containing 100 μg/mL RNase (Sigma-Aldrich, Darmstadt, Germany). Each sample (at least 10,000 cells) was analyzed using a LSRFortessa flow cytometer (BD Biosciences, Franklin Lakes, NJ, USA) and the data were processed by FCS Express 5 software (http://www.denovosoftware.com).

### RNA extraction and reverse transcription quantitative polymerase chain reaction (RT-qPCR)

After HT29 and HCT116 cells were treated with WT or FadA−/− *Fusobacterium nucleatum* at various concentrations and time periods (MOI: 1000), RNA was extracted using a Trizol kit (Invitrogen, Carlsbad, CA, USA). RNA (5 μg) was reverse transcribed to cDNA using a cDNA kit (K1622; Fermentas Inc., Ontario, CA, USA). Real-time quantitative PCR was performed using PrimeScript RT-PCR kits (TaKaRa, Shiga, Japan) and iQ5 qPCR System (Bio-Rad, Hercules, CA, USA) to quantify chk2 expression. Glyceraldehyde phosphate dehydrogenase (GAPDH) was used as an internal reference. The sequence of chk2 was: Forward (5′ → 3′): TCTCGGGAGTCGGATGTTGAG, Reverse (5′ → 3′): CCTGAGTGGACACTGTCTCTAA, and that of GAPDH was: Forward (5′ → 3′): ACGGATTTGGTCGTATTGGGCG, Reverse (5′ → 3′): CTCCTGGAAGATGGTGATGG (RiboBio Co. Ltd., Guangzhou, China). The relative mRNA expression of the target gene was calculated by the 2^-ΔΔCt^ method. The experiment was repeated 3 times.

### Production of monoclonal antibodies against FadA [31]

The mouse anti-FadA monoclonal antibody (mAb) 5G11-3G8 was produced in our laboratory. In detail, the hybridomas secreting mAb were obtained from the BALB/c mice immunized with recombinant mFadA. Antibody specific binding to FadA in enzyme-linked immunosorbent assay was performed for identification of antibodies of the desired specificity, and Western blot analysis was conducted with purified FadA proteins and *Fusobacterium nucleatum*. One of the hybridoma clones was designated as 5G11-3G8. The mAb from this clone was harvested from the serum-free culture, purified with a protein G column, and stored at a final concentration of 4 mg/mL.

### Western blot analysis

Proteins in the cell membrane, cytoplasm, and nuclei were extracted using the Compartmental Protein Extraction Kit (Millipore, Burlington, MA, USA). Standard Western blot analysis procedures were performed. Proteins were incubated with primary rabbit antibodies (Abcam, Cambridge, UK) to E-cadherin (also as CDH1, 1:1000, ab181860), phosphorylated CDH1 (1:1000, ab76319), chk2 (1:1000, ab109413), β-catenin (1:1000, ab2365), phosphorylated β-catenin (1:1000, ab81305), LaminA (1:3000, ab8984), and GAPDH (1:2500, ab9485).

### Statistical analysis

SPSS 21.0 statistical software (IBM-SPSS Statistics, Chicago, IL, USA) was used for statistical analysis. Data were expressed as mean ± standard deviation (s.d.). Data from two groups were compared using the unpaired *t* test. Data from multiple groups were compared using one-way analysis of variance (ANOVA) and Tukey’s post hoc test. Comparison of data from tumors at different time points was performed using repeated measures ANOVA and the number of cells at different time points was compared using two-way ANOVA, followed by Bonferroni post hoc test. Non-parametric Mann-Whitney U test was used for comparing two-group data that were not normally distributed. Difference were considered significant when *p* < 0.05.

## Results

### WT *Fusobacterium nucleatum* induces CRC in mice

**Fig. 1:**
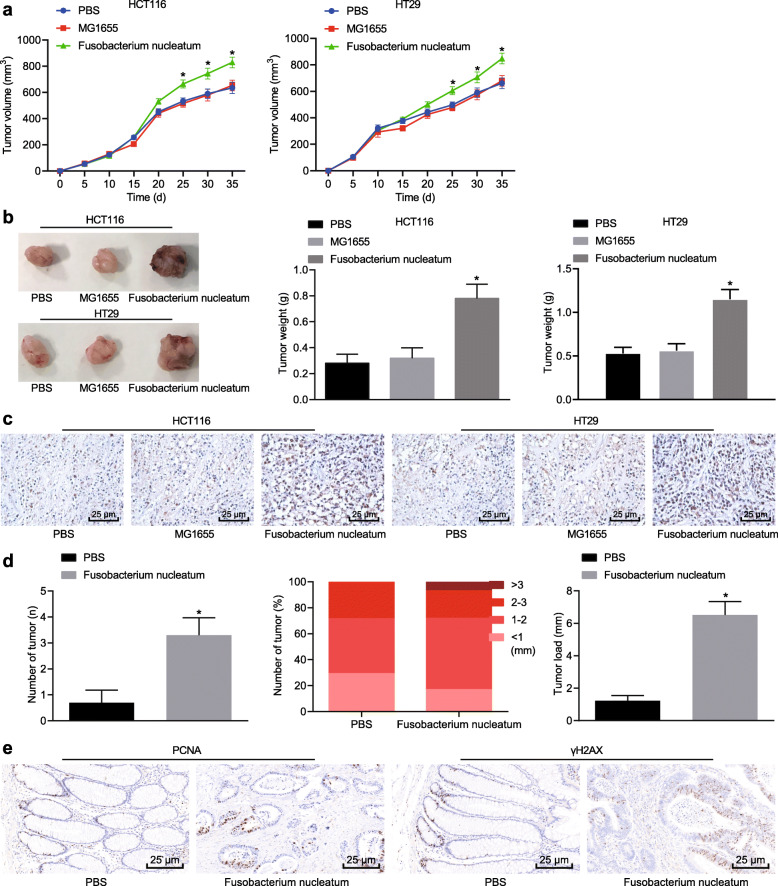
*Fusobacterium nucleatum* contributes to CRC. Male BALB/C nude mice were subcutaneously injected with HCT16 and HT29 cells treated with PBS, *Escherichia coli*, or *Fusobacterium nucleatum* to establish xenograft tumor animal models. **a** Tumor volume in HT29 and HCT116 cells. **b** Weight of tumors in nude mice formed by cells with different treatments. **c** Representative micrographs showing Ki-67 immunohistochemistry in xenograft tumor tissues (400 ×). **d** Number of tumors and tumor load in APC (Min/+) mice (*n* = 10 per group). **e** Representative micrographs showing PCNA (400 ×) and γH2AX (400 ×) immunohistochemistry. *p < 0.05 vs. PBS or *Escherichia coli*. Data are expressed as mean ± s.d. Data from multiple groups were compared using one-way analysis of variance (ANOVA) and Tukey’s post hoc test. Data comparison at different time points was performed using repeated measures ANOVA, followed by Bonferroni post hoc test. Non-parametric Mann-Whitney U test was used for comparison of data that were not normally distributed

We injected HT29 cells and HCT116 cells co-cultured for 24 h with WT *Fusobacterium nucleatum* subcutaneously into BALB/c nude mice. WT *Fusobacterium nucleatum* significantly increased tumor volume induced by HT29 and HCT116 cells when compared to PBS or *Escherichia coli* treatment (Fig. 1a). Similarly, WT *Fusobacterium nucleatum* treatment also increased tumor weight in nude mice (Fig. 1b). The cell proliferation marker Ki-67 in the xenograft tissues was increased by *Fusobacterium nucleatum* treatment as compared with PBS or *Escherichia coli* treatment (Fig. 1c). Put together, these results suggested that WT *Fusobacterium nucleatum* played a carcinogenic role in CRC.

Mice carrying *adenomatous polyposis coli* gene mutations, APC (Min/+), are susceptible to a variety of intestinal tumors. WT *Fusobacterium nucleatum* significantly increased the number of colorectal tumors, tumor size (> 3 mm) and tumor burden in APC (Min/+) mice when compared with control mice (Fig. 1d). The cell proliferation marker PCNA and DNA double-strand breaks marker γH2AX were significantly higher in CRC tissues from WT *Fusobacterium nucleatum*-treated mice (Fig. 1e), suggesting that WT *Fusobacterium nucleatum* could effectively promote the proliferation and DNA damage in colorectal epithelial cells. Altogether, these results indicated that WT *Fusobacterium nucleatum* promoted CRC growth.

### WT *Fusobacterium nucleatum* increases DNA damage in CRC cells

**Fig. 2:**
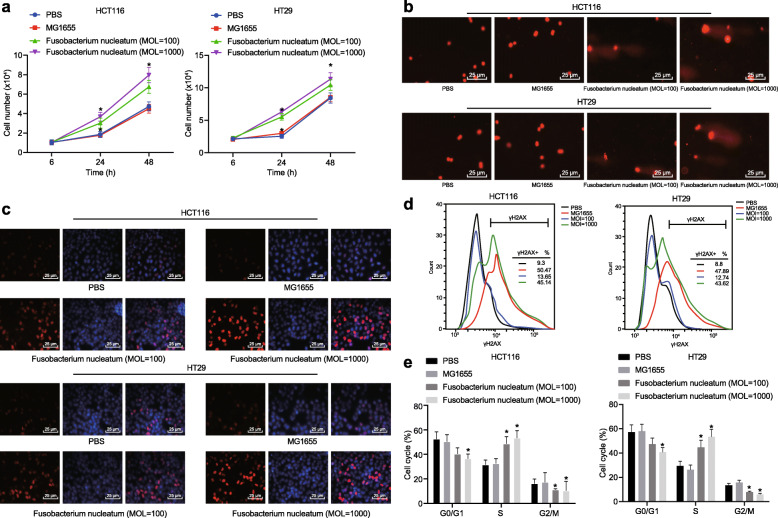
*Fusobacterium nucleatum* leads to DNA damage in CRC cells. HCT16 and HT29 cells were treated with PBS, *Escherichia coli*, or *Fusobacterium nucleatum* with different MOI. **a** The number of cells counted by hemocytometry at hour 6, 24 and 48 after treatment to detect cell proliferation. **b** Representative micrographs (400 ×) showing Comet assay. **c** Representative micrographs (400 ×) showing γH2AX immunofluorescence assay. **d** γH2AX formation determined by flow cytometry. **e** The number of cells in different cell cycle phases. *p < 0.05 vs. PBS or *Escherichia coli*. Data are expressed as mean ± s.d. Data from multiple groups were compared using one-way analysis of variance (ANOVA) and Tukey’s post hoc test. Data comparison at different time points was performed using repeated measures ANOVA, followed by Bonferroni post hoc test. Each experiment was repeated three times

In order to explore the mechanism of induction of CRC by *Fusobacterium nucleatum*, cells were co-cultured with WT *Fusobacterium nucleatum*, *Escherichia coli* (MOI: 1000 or 100:1) or PBS for 24 h. WT *Fusobacterium nucleatum* significantly promoted the growth of HT29 and HCT116 cells when compared to PBS or *Escherichia coli* (Fig. 2a). Moreover, the Comet assay showed that WT *Fusobacterium nucleatum* significantly enhanced DNA damage in HT29 and HCT116 cells as compared with PBS or *Escherichia coli* treatment (Fig. 2b). Similarly, the formation of γH2AX, a marker for DNA damage, was significantly increased in *Fusobacterium nucleatum*-treated cells when compared to PBS- or *Escherichia coli*-treated cells, as determined by the immunofluorescence assay (Fig. 2c) or flow cytometry (Fig. 2d). *Fusobacterium nucleatum* also arrested more cells in the S phase when compared to PBS and *Escherichia coli* (Fig. 2 e). These results suggested that *Fusobacterium nucleatum* induced DNA damage in CRC cells.

### *Fusobacterium nucleatum* elevates DNA damage in CRC cells via FadA

**Fig. 3:**
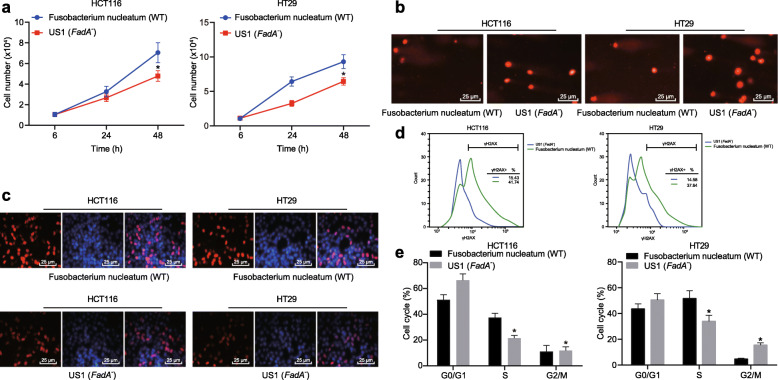
*Fusobacterium nucleatum* induces DNA damage in CRC cells via FadA. HCT16 and HT29 cells were treated with WT or FadA−/− *Fusobacterium nucleatum* (MOI: 1000). **a** The number of cells counted by hemocytometry at at hour 6, 24 and 48 after treatment to detect cell proliferation. **b** Representative micrographs showing Comet analysis. **c** Representative micrographs (400 ×) depicting the γH2AX immunofluorescence assay. **d** γH2AX formation as determined by flow cytometry. **e** The number of cells in different cell cycle phases *p < 0.05 vs. WT *Fusobacterium nucleatum*. Data are expressed as mean ± s.d. Data from two groups were compared using the unpaired t test. Data comparison at different time points was performed using repeated measures ANOVA, followed by Bonferroni post hoc test. Each experiment was repeated three times

With an aim to investigate whether the mechanism of DNA damage induced by *Fusobacterium nucleatum* was related to FadA, we constructed FadA−/− *Fusobacterium nucleatum*. We treated HT29 and HCT116 cells with WT *Fusobacterium nucleatum* and FadA−/− *Fusobacterium nucleatum* (MOI = 1000). Cell growth in FadA−/− *Fusobacterium nucleatum*-treated cells was reduced as compared to WT *Fusobacterium nucleatum* (Fig. 3a). DNA damage, as determined by Comet assay, was reduced in FadA−/− *Fusobacterium nucleatum*-treated cells when compared to WT *Fusobacterium nucleatum* (Fig. 3b). FadA−/− also significantly reduced γH2AX formation, as determined by the immunofluorescence assay (Fig. 3c) or flow cytometry (Fig. 3d). FadA−/− *Fusobacterium nucleatum* treatment significantly reduced the number of cells in S phase when compared to treatment with WT *Fusobacterium nucleatum* (Fig. 3e). These results suggested that DNA damage in CRC cells caused by *Fusobacterium nucleatum* occurred through modulation of FadA.

### FadA−/− reduces the activation of E-cadherin/β-catenin and chk2 in CRC cells

**Fig. 4:**
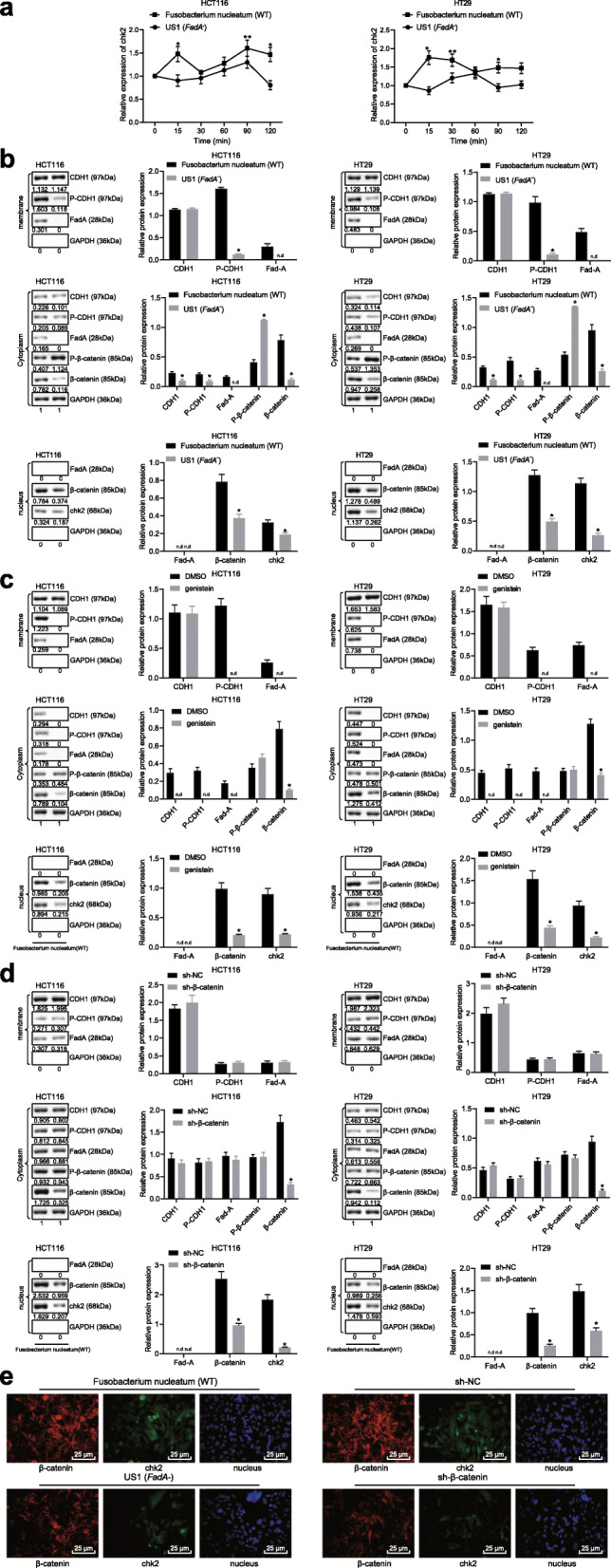
FadA regulates E-cadherin, β-catenin, and chk2 expression in CRC cells. **a** Expression of chk2 in HCT116 and HT29 cells after treatment with WT or FadA−/− *Fusobacterium nucleatum* (MOI: 1000) for different time periods. **b** Protein expression of FadA, E-cadherin, β-catenin, and chk2 in HCT116 and HT29 cells treated with WT or FadA−/− *Fusobacterium nucleatum* (MOI: 1000) for 2 h. **c** Protein expression of FadA, E-cadherin, β-catenin, and chk2 in HCT116 and HT29 cells treated with genistein for 1 h and then with WT or FadA−/− *Fusobacterium nucleatum* (MOI: 1000) for 2 h. PTK inhibitor genistein inhibits all FadAc-activated functions. **d** Protein expression of FadA, E-cadherin, β-catenin, and chk2 in β-catenin-knockdown HCT116 and HT29 cells treated with WT or FadA−/− *Fusobacterium nucleatum* (MOI: 1000) for 2 h. **e** β-catenin nucleation and chk2 expression in HCT116 cells after different treatments determined by a confocal microscopy (400 ×). * *p* < 0.05 or ** *p* < 0.01 vs. WT *Fusobacterium nucleatum*. Data are expressed as mean ± s.d. and n.d. stands for no data. Data comparison at different time points was performed using repeated measures ANOVA, followed by Bonferroni post hoc test. Non-parametric Mann-Whitney U test was used for data that were not normally distributed

Elucidating the relationship between FadA and chk2, we found that expression of chk2 was decreased by FadA−/− *Fusobacterium nucleatum* when compared to WT *Fusobacterium nucleatum* in CRC cells (Fig. 4a). It has been previously reported that FadA-regulated E-cadherin/β-catenin promoted cell growth in CRC [26]. Therefore, we determined the expression of E-cadherin (CDH1) and β-catenin. FadA−/− decreased phosphorylation and internalization of E-cadherin (CDH1) on the cell membrane (Fig. 4b). FadA−/− also decreased internalization of β-catenin, leading to reduced chk2 expression. Then, we studied the role of protein tyrosine kinase by using its inhibitor genistein. Genistein treatment not only prevented the phosphorylation and internalization of E-cadherin, but also prevented FadA from binding to the cell membrane and internalizing, leading to decreased expression of β-catenin and chk2 (Fig. 4c). Besides, β-catenin knockdown did not affect the binding of FadA on E-cadherin on the cell membrane, the phosphorylation and internalization of E-cadherin, but reduced chk2 expression (Fig. 4d). Furthermore, using confocal microscopy, we showed *Fusobacterium nucleatum* promoted the entry of β-catenin to the nucleus and increased chk2 expression, both of which were reduced by β-catenin knockdown (Fig. 4e).

### FadA upregulates E-cadherin/β-catenin activation and chk2 to induce DNA damage of CRC cells

**Fig. 5:**
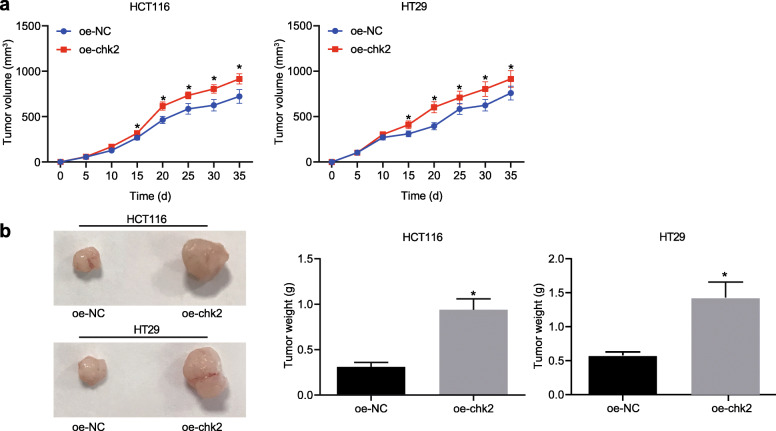
Overexpression of chk2 induces tumor growth in nude mice. Male BALB/C nude mice were subcutaneously injected with chk2-overexpressed HCT16 and HT29 cells to establish xenograft tumor animal models (*n* = 5 per group). **a** Tumor volume in nude mice. **b** Tumor weight in nude mice. * *p* < 0.05 vs. oe-NC. Data are expressed as mean ± s.d. Data from two groups were compared using the unpaired t test. Data comparison at different time points was performed using repeated measures ANOVA, followed by Bonferroni post hoc test. Each experiment was repeated three times

**Fig. 6:**
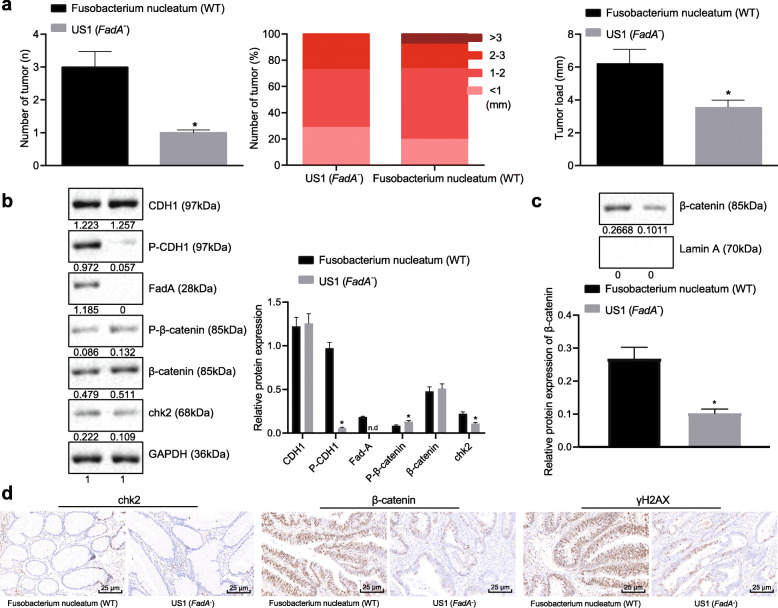
FadA up-regulates E-cadherin/β-catenin activation and chk2 to induce DNA damage in CRC cells. **a** Tumor number, size, and load in APC^Min/+^ mice on 12th week after treatment with WT or FadA−/− *Fusobacterium nucleatum*. **b** Protein expression of FadA, E-cadherin/β-catenin, and chk2 in CRC tissue determined by Western blot analysis. **c** Protein expression of β-catenin in the nucleus. **d** Representative micrographs showing β-catenin (400 ×), chk2 protein (400×), and γH2AX (400 ×) immunohistochemistry in CRC tissues. Data are expressed as mean ± s.d. Data from two groups were compared using the unpaired t test

First of all, in order to prove that the up-regulation of chk2 could aggravate DNA damage in CRC cells, we overexpressed chk2 in CRC cells. The results demonstrated that chk2 overexpression caused DNA damage in CRC cells (Supplementary Fig. 1A, B), as shown by the Comet assay and immunofluorescence assay. These cells were injected to nude mice. We found that chk2 overexpression increased tumor volume (Fig. 5a) and weight (Fig. 5b) in vivo. In addition, nude mice treated with FadA−/− *Fusobacterium nucleatum* had reduced number, size, and load of tumors in the colon when compared to mice treated with WT *Fusobacterium nucleatum* (Fig. 6a). In CRC tissue taken from FadA−/− *Fusobacterium nucleatum*-treated mice, FadA expression was absent, phosphorylation of E-cadherin and expression of chk2 were decreased, while phosphorylation of β-catenin increased (Fig. 6b). In addition, FadA−/− decreased the expression of β-catenin in the nucleus (Fig. 6c). As shown in immunohistochemical staining, FadA−/− also reduced the expression of β-catenin, chk2 protein, and γH2AX in CRC tissues (Fig. 6d). These results showed that FadA was involved in up-regulation of chk2 and increased DNA damage in CRC by activating the E-cadherin/β-catenin pathway.

## Discussion

**Fig. 7:**
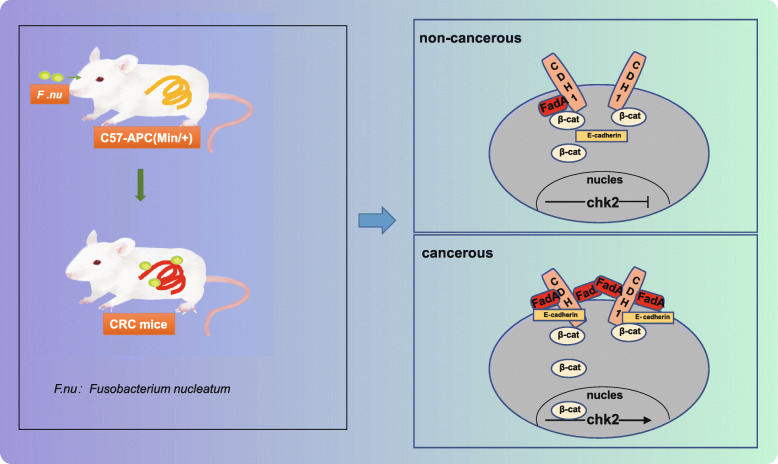
Schematic diagrams showing the proposed molecular pathway of *Fusobacterium nucleatum*-induced FadA-dependent DNA damage and development of CRC

*Fusobacterium nucleatum* has been implicated in CRC, but the underlying molecular mechanisms remain to be understood [26]. In this study, we found that *Fusobacterium nucleatum* promoted the progression of CRC in a mouse model and was related to DNA damage in CRC cells. Secondly, FadA knockout normalized the effects of *Fusobacterium nucleatum* on CRC. Thirdly, chk2 overexpression increased DNA damage and the growth of CRC, and lastly, FadA knockout reduced E-cadherin pathway and the expression of chk2. Based on these results, we proposed that FadA in *Fusobacterium nucleatum* bound to and activated the E-cadherin/β-catenin pathway, leading to increased chk2 expression, DNA damage, and progression of CRC (Fig. 7).

Our initial experiments showed that *Fusobacterium nucleatum* caused CRC progression in APC (Min/+) mice, results that were similar to other studies [32–34]. *Fusobacterium nucleatum* infection in the colon has been implicated as another environmental risk factor for CRC [35–37], in addition to many other environmental and non-modifiable risk factors reported previously [6, 9–11]. Moreover, using this validated APC (Min/+) model, we further demonstrated that FadA in *Fusobacterium nucleatum* was critical for DNA damage and CRC progression. FadA, a novel adhesin unique to oral *Fusobacteria*, is required for *Fusobacterium nucleatum* to bind to and invade epithelial cells, and may therefore, assume a critical role in *Fusobacteria* colonization of a host [38]. Our results were also aligned with those of previous studies showing the involvement of FadA in *Fusobacterium nucleatum*-related CRC [26, 27]. These studies also support our results showing FadA may work through the activation of E-cadherin/β-catenin pathway to facilitate CRC progression.

Our subsequent results confirmed and verified the involvement of FadA-activated E-cadherin/β-catenin pathway in the development of CRC. A previous study showed that FadA bound to E-cadherin in CRC cells [26]. The activation, as well as the inhibition, of the E-cadherin/β-catenin pathway has been shown to be involved in multiple cancers including renal and liver cancers [39–41]. The E-cadherin/β-catenin pathway, therefore, has been proposed to be a potential target for cancer therapy because of its role in regulating genes or mediators involved in cancer development and progression [42–44]. In a related finding, Zhao et al. demonstrated the involvement of the E-cadherin/β-catenin pathway activation in CRC development [45]. As an interpretation, these results suggest that an inhibitor of the E-cadherin/β-catenin pathway may be used to potentially treat *Fusobacterium nucleatum*-related CRC.

We also found that FadA enhanced E-cadherin/β-catenin activation to upregulate chk2 in turn, thereby inducing DNA damage in CRC cells. Chk2 has been implicated in other cancers as well, such as breast cancer [46]. In CRC, the involvement of chk2 is also well-documented [47–50]. Therefore, results from this study added to the knowledge suggesting chk2 involvement in the specific *Fusobacterium nucleatum*-related form of CRC. In particular, chk2 was responsible for increased DNA damage in CRC cells and increased tumor growth in vivo. Previous studies also demonstrated that chk2-mediated DNA damage is important in the progression of CRC [51, 52]. Furthermore, our study also demonstrated that DNA damage in CRC cells may be due to delayed cell cycle process, similar results having been noted previously showing chk2-mediated DNA damage in CRC [53]. Collectively, these data implying the involvement of chk2 suggest potential therapeutic targets for the treatment of CRC in different stages [54, 55]. When activated, chk2 is known to inhibit CDC25C phosphatase, preventing entry into mitosis, and stabilizing the tumor suppressor protein p53, leading to cell cycle arrest in G1 [56]. In addition, it has also been reported that chk2 interacts with phosphorylated BRCA1, allowing BRCA1 to restore survival after DNA damage [57]. These findings can trigger an exploration of the activated downstream mediators of chk2 in the future.

## Conclusion

In conclusion, this study provides evidence that chk2 may be a newly discovered mediator in DNA damage and progression of *Fusobacterium nucleatum*-induced, E-cadherin/β-catenin pathway-related CRC. Chk2 and the checkpoint response may warrant further study as therapeutic targets relevant to different stages of CRC. However, the animal model used in this study has not been fully characterized and therefore may not mimic all aspects of human *Fusobacterium nucleatum*-related CRC. Secondly, although our results demonstrated that chk2 expression was decreased by FadA−/− and chk2 overexpression increased DNA damage and CRC progression, there was no true causal relationship between FadA and chk2 established in this study. And lastly, the effect of FadA overexpression was not studied in this study due to the lack of suitable research tools.

## Supplementary information

**Additional file 1: Supplemental Fig. 1.** The effect of chk2 overexpression on DNA damage in CRC cells. Representative micrographs showing (A) Comet assay (400 ×) and (B) γH2AX immunofluorescence assay (400 ×). At least three independent experiments were conducted.

## Data Availability

The datasets generated/analyzed during the current study are available.

## Abbreviations

CRC: Colorectal cancer
APC: Adenomatous polyposis coli
Chk2: Checkpoint kinase 2
PBS: Phosphate buffered saline
FBS: Fetal bovine serum
NC: Negative control
BSA: Bovine serum albumin
PCR: Reverse transcription quantitative polymerase chain reaction
s.d: Standard deviation
ANOVA: Analysis of variance
CDH1: Cadherin
